# Tuning friction and slip at solid-nanoparticle suspension interfaces by electric fields

**DOI:** 10.1038/s41598-019-54515-1

**Published:** 2019-12-09

**Authors:** B. Acharya, C. M. Seed, D. W. Brenner, A. I. Smirnov, J. Krim

**Affiliations:** 1 Department of Physics, North Carolina State University, Raleigh, NC 27695 USA; 2 Department of Materials Sci. and Eng., North Carolina State University, Raleigh, NC 27695 USA; 3 Department of Chemistry, North Carolina State University, Raleigh, NC 27695 USA

**Keywords:** Nanoparticles, Batteries

## Abstract

We report an experimental Quartz Crystal Microbalance (QCM) study of tuning interfacial friction and slip lengths for aqueous suspensions of TiO_2_ and Al_2_O_3_ nanoparticles on planar platinum surfaces by external electric fields. Data were analyzed within theoretical frameworks that incorporate slippage at the QCM surface electrode or alternatively at the surface of adsorbed particles, yielding values for the slip lengths between 0 and 30 nm. Measurements were performed for negatively charged TiO_2_ and positively charged Al_2_O_3_ nanoparticles in both the absence and presence of external electric fields. Without the field the slip lengths inferred for the TiO_2_ suspensions were higher than those for the Al_2_O_3_ suspensions, a result that was consistent with contact angle measurements also performed on the samples. Attraction and retraction of particles perpendicular to the surface by means of an externally applied field resulted in increased and decreased interfacial friction levels and slip lengths. The variation was observed to be non-monotonic, with a profile attributed to the physical properties of interstitial water layers present between the nanoparticles and the platinum substrate.

## Introduction

Pathways to achieve climate stabilization consistently require a diverse mix of technologies, with energy efficiency being the largest contributor, ranging from 35–40% in virtually all proposed scenarios^1,2^. Reductions in frictional energy losses and improved lubrication methodologies are key to such energy savings^3^. Traditional lubricants for present day oil-based lubricant technologies are, however, linked to a wide range of environmental, health, and safety hazards associated with their leakage, handling, and disposal^4^. Their expanded use, while reducing carbon emissions, adds additional toxins to the environment. Development of environmentally friendly lubricants, enabled by recent progress in nanostructured materials that, for example, allow water to be used as the working fluid in place of oil, is therefore of major interest^5^, as well as tribological properties of water itself^6^. Nanoparticles are known to impact friction levels at both the nanoscale and macroscale when added to liquid lubricants, including water^7–9^. Reports of both reductions and increases in friction by 10–70% can be found in the literature^10–13^, with performance being closely linked to nanoparticle size, concentration, electrical charge, and the surface treatment employed to prevent particle agglomeration^7,13^. A fundamental understanding of the physical and chemical processes that govern the role of nanoparticles in such applications has yet however to be established.

We focus here on Al_2_O_3_ and TiO_2_ nanoparticles, which have widespread technological applications on account of their ease of mass production and environmental friendliness^14,15^. Al_2_O_3_ nanoparticles are widely used in surface coatings, ultra-fine polishing, and as additives to heat transfer fluids and oil or water-based lubricants^16–18^. TiO_2_ nanoparticles are in even broader use in applications ranging from photocatalysis and photovoltaics to water-splitting owing to the wide-band-gap nature of this semiconductor^19–22^. One area of particular interest involves energy storage devices and fuel cells^22^. Electric double layer capacitors have proven potential for use in these power management systems, and nanostructured TiO_2_ electrodes formed from nanoparticles, nanotubes and/or nanospindles represent a highly promising approach^22–24^. Progress in these applications relies on knowledge of how the nanoparticles dispersed in a suspension interact at the nanoscale with the solid-liquid interfaces that they are in a contact with.

**Figure 1:**
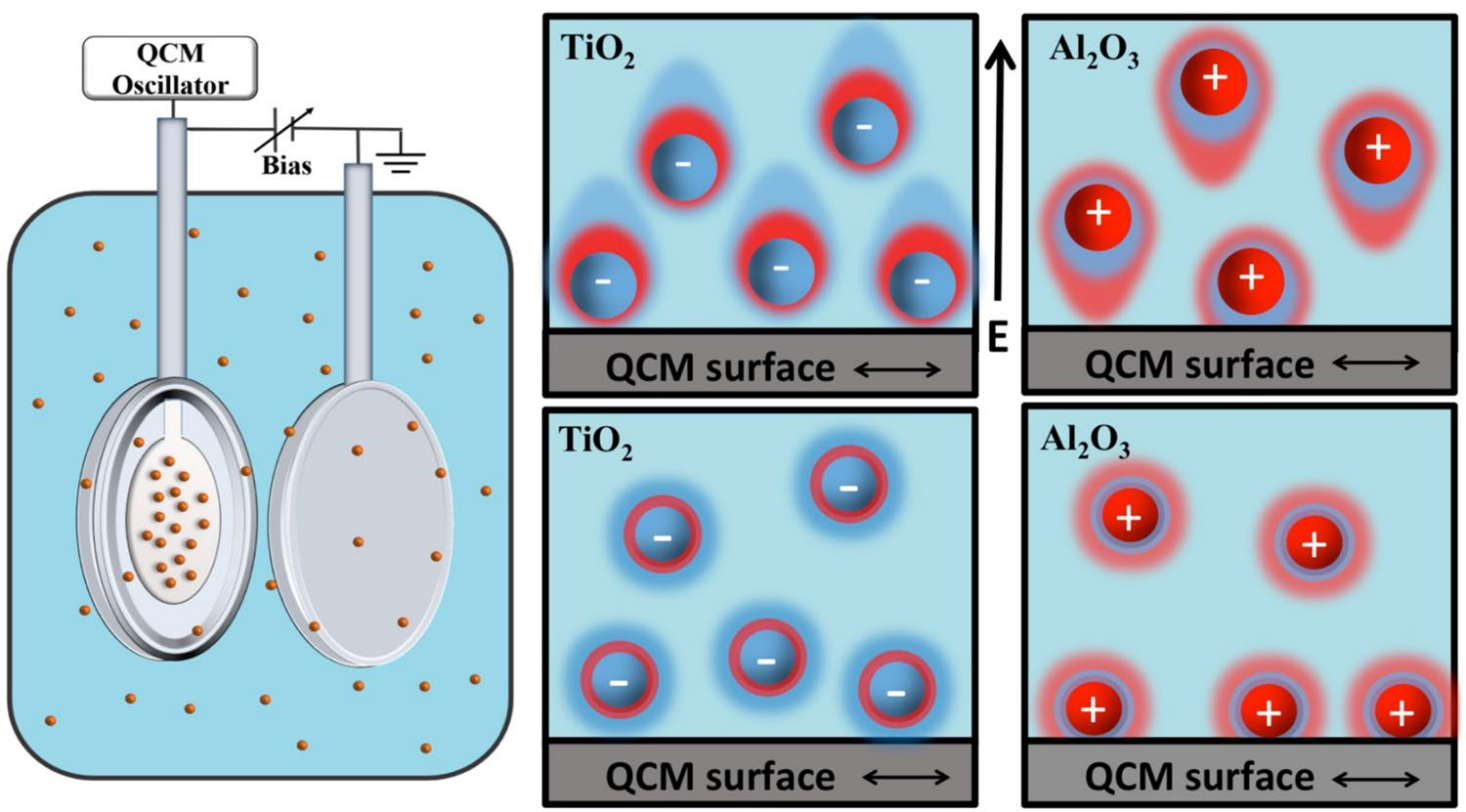
Schematic of the apparatus and an example of the response of positively charged Al_2_O_3_ nanoparticles and negatively charged TiO_2_ nanoparticles to a positive bias voltage applied to the Pt QCM electrode. Positive bias voltages repel (attract) the Al_2_O_3_ (TiO_2_) nanoparticles away from (towards) the surface, changing both the number of nanoparticles near the surface as well as the location of the hydrodynamic slip plane and the electrical double layer. Interfacial frictional drag levels are highly sensitive to both effects.

The Quartz Crystal Microbalance (QCM) technique is exceptionally sensitive to changes in slippage and mass uptake at interfaces, and is an ideal tool for studies of interfacial properties of molecularly thin materials when immersed into liquids^25–34^. Interpretation of the data can, however, be challenging for complex interfaces^25^. We employed QCM here to study the interfacial properties of planar platinum surfaces immersed in aqueous suspensions of negatively or positively charged nanoparticles composed of TiO_2_ and Al_2_O_3_, respectively. We then applied external electric fields to induce electrophoretic forces for repositioning of the nanoparticles in the direction perpendicular to the QCM surfaces (Fig. 1). In addition to inducing electrophoretic motion, electric fields deform the double layers and location of hydrodynamic slip planes, induce dipole moments, and impact nanoparticle self-assembly and ordering^35–37^. The ability to reposition the nanoparticles allows for both control of the interfacial properties and also provides a means to assess the applicability of theories employed to analyze the results. Our motivations were twofold: (1) to explore the degree to which interfacial friction levels associated with the presence of nanoparticles could be tuned by an external electric field, and (2) to explore active control of nanoparticles as a probe of solid-liquid interfacial properties.

## Materials and Methods

### Materials

Aqueous suspensions of ~20 wt% anatase TiO_2_ (titania) and γ-Al_2_O_3_ (alumina) nanoparticles were obtained from US Research Nanomaterials (stock numbers US7071 and US7030; Houston, TX 77084, USA). These stock solutions were further dispersed in deionized water to obtain the desired concentrations. Measurements were conducted within 48 h of preparing the suspensions. No nanoparticle aggregation was observed during this period. Some sedimentation was, however, detected for suspensions left overnight that could be reversed by stirring and sonication. The suspensions were therefore stirred and sonicated for 10–15 min immediately before each experimental measurement, and all measurements were completed within 60 min of this procedure.

The values of the physical properties of the suspensions and nanoparticles employed in this study are listed in Table 1. The viscosity is estimated by Einstein’s equation^38^ ${\eta }_{nf}={\eta }_{0}(1+2.5\varphi ),$ where *η*_0_ is the viscosity in the absence of nanoparticles and *η*_*nf*_ is the viscosity of a suspension with volume fraction concentration φ of nanoparticles. Surface tension values for the suspensions were obtained from ref. ^39^. The total surface charge per particle *q*_p_ was estimated using the approach described in ref. ^40^. At concentrations of 0.67 wt% the particle densities in the TiO_2_ and Al_2_O_3_ suspensions were respectively 1.2 × 10^20^ m^−3^ and 4.7 × 10^19^ m^−3^, and the average particle-to-particle distances were respectively 4.05 × 10^−7^ m and 5.54 × 10^−7^ m. The average mass of the particles were respectively 1.42 × 10^−16^ g and 5.59 × 10^−17^ g and the mass per unit area of monolayers of the NP attached in close-packed lattice would respectively be *ρ*_2_ = 1.02 × 10^−5^ g/cm^2^ and *ρ*_2_ = 7.17 × 10^−6^ g/cm^2^.

**Table 1:** Physical properties of water and 0.67% nanoparticle suspensions at 23 °C.

	Water and 0.67 wt% nanoparticle suspension properties					Nanoparticle properties			
	Viscosity mpoise	Density g/cm^3^	Surface tension dyne/cm	pH	Volume fraction wt %	Density g/cm^3^	Zeta potential mV	Radius nm	Charge 10^–18^ C
Water	9.321	0.99759	72.3	6.7					
TiO_2_	9.3568	1.00367	75.5	7.7	0.16	4.23	−32.7 ± 0.8	20	−28.2
Al_2_O_3_	9.3517	1.00282	75.5	4.5	0.14	3.95	+60.8 ± 0.8	15	+51.2

The QCM crystals (Fil-Tech Inc., Boston, MA) were 5 MHz AT-cut (Temperature compensated transverse shear mode type A) quartz, 1” in diameter with liquid-facing Pt surface electrodes that were ½” in diameter and rear-facing electrodes of ¼” in diameter. The Pt electrodes were deposited atop Ti adhesive layers on overtone-polished surfaces. A QCM’s sensitivity zone is characterized by the shear-wave penetration depth into the liquid given by *δ* = (2*η*/ω*ρ*)^1/2^. For the crystals employed here the penetrations depths for all fluids are close to 244 nm^41^.

### Methods

QCM data were recorded with a QCM100 system (Stanford Research Systems, Sunnyvale, CA, USA) employing a Teflon sample holder that exposed one side of the QCM to the surrounding liquid^42^. QCM resonant frequency *f* and conductance voltage *V*_*c*_ are the measured output signals. The “motional resistance”, or effective additional electrical resistance to the circuit *δR* upon immersion of a QCM crystal in a liquid is directly proportional to shifts in the oscillator’s inverse quality factor *δ*(*Q*^*−1*^) with a proportionality factor of the order of ~10^−6^, as described in detail in ref. ^29^. Shifts in frequency and inverse quality factor *Q*^*−1*^ respectively reflect the mass of the material dragged along with the oscillatory motion and the frictional forces impeding the QCM’s motion. For exposure of one oscillating electrode of the QCM to mass loading and/or a surrounding fluid they are related to the acoustic impedance ${\mathscr{Z}}={\mathscr{R}}-i{\mathscr{X}}$ acting on the electrode as^43^:

1 $$\delta (\frac{1}{Q})=\frac{2 {\mathcal R} }{\pi \sqrt{{\rho }_{q}{\mu }_{q}}};\,\delta f=-\frac{f{\mathscr{X}}}{\pi \sqrt{{\rho }_{q}{\mu }_{q}}}$$

where *ρ*_q_ = 2.648 g cm^−3^ and *μ*_q_ = 2.947 × 10^11^ g cm^−1^s^−2^ are respectively the density and the shear modulus of quartz. Other parameters commonly employed in the literature to represent the system dissipation are the unitless damping factor *D*_f_, the unitless dissipation *D*, and the half bandwidth at half maximum *Γ* that has units of Hz. These parameters are mutually related as $\delta ({Q}^{-1})=(\frac{2}{f})\delta \varGamma =(\frac{1}{\pi })\delta {D}_{f}=\delta D.$

Changes in *f* and *V*_*c*_. were recorded for QCM crystals mounted in sample holders under ambient conditions and then immersed in DI water. TiO_2_ or Al_2_O_3_ nanoparticles from the stock suspensions were added next while continuing to record changes in *f* and *V*_*c*_. Changes in the conductance voltage were converted to those in motional resistance according to the relation $R={{\rm{10}}}^{(4-{V}_{c}/5)}-{\rm{75}}$^42^. Data were recorded for nanoparticle concentrations ranging from 0 to 1 wt% in equal intervals of 1/6 wt % first without any external fields (*i.e*., zero field conditions), and next in constant (both attractive and repulsive) or slowly varying electric fields at fixed nanoparticle concentrations of 0.67 wt%. For the latter experiments a second identical QCM mounted in the identical Teflon holder was positioned within the suspension at a distance of 1.5 cm to serve as a grounded counter electrode (Fig. 1). This arrangement allowed the two electrode surfaces to be kept parallel to each other and facilitated uniformity of the electric field in the region between two electrodes. The arrangement was selected for its convenience. A suitably shaped platinum counter electrode could also have been utilized. A bias voltage was then applied to the sensing electrode while maintaining the counter electrode at ground. The applied potential of ±1.5 V (peak or constant) produced an electric field of ±100 N/C in the region between the electrodes. Measurements were carried out at least 5 times each for the case of static electric fields and for at least 5 cycles for the slowly alternating electric field.

Contact angle measurements were performed in air on the QCM Pt electrodes via a drop shape analysis method^44^. In this method, the contact angle *θ* is defined as tan(*θ*/2) = *h/d*, where *h* and *d* are respectively the height and the diameter of sessile droplets deposited onto the surface of interest. AFM measurements were recorded to characterize rms surface roughness levels *σ*(*L*) as a function of the lateral scan size *L*, generating values for the self-affine roughness exponent and the lateral correlation lengths^29^.

## Theoretical Analysis Approaches

### QCM data analysis

**Figure 2:**
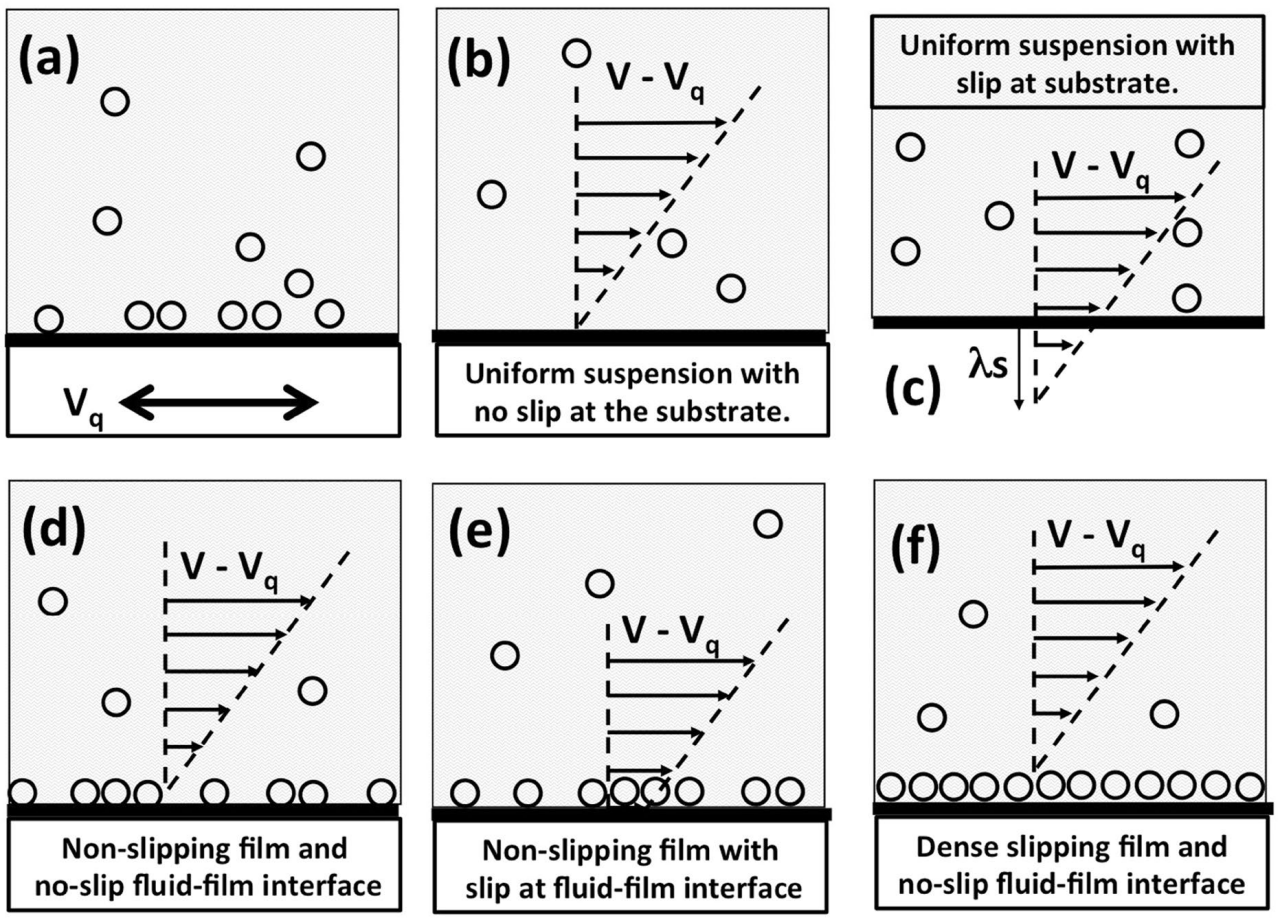
(**a**) Schematic of transverse shear motion of QCM motion with oscillation amplitude V_q_ immersed in a nanoparticle suspension along with the various theoretical frameworks employed to analyze the QCM data. The models fall into three general categories: “Bulk” (**b**,**c**) uniform suspensions, “Electrolyte” (**d**,**e**) slippage occurs at the boundary of a non-slipping surface layer with the fluid, and “Adsorbed film”(**f**) slippage occurs at the boundary of a dense adsorbed film with the substrate.

QCM data were analyzed using available theories that considered several models for film and/or bulk phases in contact with the oscillating electrode under slip and non-slip boundary conditions (Fig. 2)^27^. These idealized limiting cases reveal scenarios for starting conditions that could be used for more detailed theoretical modeling. Intermediate cases, for example systems falling between Fig. 2c,e, would require more extensive computational methods, as analytic solutions have not been reported in the literature.

Figure 2 depicts QCM motion with velocity amplitude *V*_q_ immersed in suspensions (a) along with various slip conditions displaying vectors that represent the difference in the velocity amplitudes of the fluid with the QCM electrode (b–f). A no-slip boundary condition corresponds to *V* − *V*_*q*_ = 0 at the electrode surface. Slippage is characterized by a non-zero value of *V(t) – V(t)*_*q*_ at the surface electrode and a slip length *λ*_*s*_, which is distance below the surface that the velocity extrapolates to zero (Fig. 2c).

For a no-slip boundary condition and in the thin film limit, the acoustic impedance ${{\mathscr{Z}}}_{2}={{\mathscr{R}}}_{2}-i{{\mathscr{X}}}_{2}$ of an adsorbed film on a QCM electrode with mass per unit area *ρ*_2_ is purely imaginary:^45^

2 $${{\mathscr{R}}}_{2}=0\,\,{{\mathscr{X}}}_{2}={\rho }_{2}\omega ,$$

Slippage of the film in response to the oscillatory motion of the electrode is treated mathematically by introducing an interfacial friction coefficient *η*_2_ defined by *F*_*f*_*/A* = *−η*_2_*(V* − *V*_*q*_) and related a characteristic slip time as *τ*  = *ρ*_2_*/η*_2_^46^. The real and imaginary components then become:^46^

3 $${{\mathscr{R}}}_{2}=\frac{{\rho }_{2}{\omega }^{2}\tau }{1+{\omega }^{2}{\tau }^{2}}\,\,{{\mathscr{X}}}_{2}=\frac{{\rho }_{2}\omega }{1+{\omega }^{2}{\tau }^{2}},$$

which reduce to the Eq. (2) expressions for the no-slip boundary condition by setting *τ* = 0.

For a QCM immersed in a bulk fluid with no film adsorption and no-slip boundary conditions, *V* = *V*_*q*_, and the real and imaginary components of the acoustic impedance are equal and related to the fluid properties as:^32^

4 $${ {\mathcal R} }_{fluid}={{\mathscr{X}}}_{fluid}=\sqrt{\pi \rho \eta \,f},$$

where *ρ* and *η* are respectively the bulk fluid density and dynamic viscosity. Boundary slip is incorporated into the model by inserting the velocity of the monolayer adjacent to the surface obtained from the friction law *F*_*f*_/*A* = *−η*_2_*(V* − *V*_*q*_) in place of the no-slip condition *V* = *V*_*q*_:^26,27^

5 $${\rho }_{2}\frac{d}{dt}{V}_{f}(t)=-\,{\eta }_{2}[V(t)-{V}_{q}(t)].$$

The acoustic impedance is obtained by solving the system equations of motion via Eq. (5) and after some rearrangement of terms it can be expressed as:^27,33^

6 $$\begin{array}{ccccc}{\mathscr{R}} & = & \sqrt{\pi \rho \eta \,f} & \cdot  & [\frac{1+2a}{{(1+a)}^{2}+{a}^{2}}]\\ {\mathscr{X}} & = & \sqrt{\pi \rho \eta f} & \cdot  & [\frac{1}{{(1+a)}^{2}+{a}^{2}}],\end{array}$$

where *a* is the ratio of the slip length *λ*_s_ to the penetration depth *δ* of the oscillations in a liquid. For fluids with uniform density the parameter *a* is related to *η*_2_ as *λ*_*s*_ = *η/η*_2_. Other parameters commonly employed in the literature to represent the boundary slip of a liquid are the slip parameter *s* = *1/η*_2_, which has units of cm^2^s/g, and the parameter $\,\chi =1/\tau$, which has units of s^−1^.

QCM data were then compared to “bulk suspension”, “electrolyte”, and “adsorbed film” models for the acoustic impedance in the idealized limiting cases discussed above. The “bulk suspension” model has no film phase and predicts Eq. (4) or Eq. (6) respectively for no-slip and slip conditions. The “electrolyte” model incorporates both a film and bulk phase, but allows the slip to occur only at the upper boundary of the film with the fluid phase (Fig. 2e):^33^

7 $$\begin{array}{ccccc}{\mathscr{R}} & = & \sqrt{\pi \rho \eta f} & \cdot  & [\frac{1+2a}{{(1+a)}^{2}+{a}^{2}}]\\ {\mathscr{X}} & = & \sqrt{\pi \rho \eta f} & \cdot  & [\frac{1}{{(1+a)}^{2}+{a}^{2}}]+\omega {\rho }_{2},\end{array}$$

In the case of a no-slip boundary condition at the film-fluid boundary, *a* = 0 and the response reduces to the sum of the separate non-slipping film and bulk contributions, Eqs. (2) and (4).

The “adsorbed film” model allows for the slippage at the boundary of the film with the QCM electrode. Mistura *et al*. considered the case of a dense thin film in a contact with a low density vapor phase and obtain expressions for the combined slipping film plus the vapor phases^34^. While this model was not originally developed for the case of immersion in a liquid, it is potentially applicable to the case of nanoparticles forming a dense compact phase that slips at the boundary with the substrate. The total system impedance in this model includes a contribution from the frictional energy dissipation at the interface parameterized by *η*_2_. In the limit where the adsorbed film thickness is much less than the penetration depth, *d* ≪ δ*,* and the bulk acoustic impedance $\sqrt{\pi f\eta \rho }$ of the surrounding fluid is far less than that of the film phase, the acoustic impedance components are described by:

8 $$\begin{array}{ccccc}\frac{{{\mathscr{R}}}_{exp}}{{{\mathscr{R}}}_{exp}^{2}+{{\mathscr{X}}}_{exp}^{2}} & = & \frac{{{\mathscr{R}}}_{v}}{{{\mathscr{R}}}_{v}^{2}+{(\omega {\rho }_{film}d+{{\mathscr{X}}}_{v})}^{2}} & + & \frac{1}{{\eta }_{2}}\\ \frac{{{\mathscr{X}}}_{exp}}{{{\mathscr{R}}}_{exp}^{2}+{{\mathscr{X}}}_{exp}^{2}} & = & \frac{\omega {\rho }_{film}d+{{\mathscr{X}}}_{v}}{{{\mathscr{R}}}_{v}^{2}+{(\omega {\rho }_{film}d+{{\mathscr{X}}}_{v})}^{2}}\end{array}$$

The mass per unit area of the film in Eq. (8) is written as *ρ*_2_ = *ρ*_*film*_*d*, where *d* is the thickness and *ρ*_*film*_ is the three dimensional density of the film phase. The measured values of the acoustic impedance can be used to solve for *ωρ*_*film*_*d*, which can be substituted into the left-hand side of Eq. (8) to obtain the interfacial viscosity *η*_2_ and the slip length *λ*_*s*_ = *η*/*η*_2_^46,47^. For the analysis employed here, we set ${ {\mathcal R} }_{v}\equiv { {\mathcal R} }_{fluid};{{\mathscr{X}}}_{v}\equiv {{\mathscr{X}}}_{fluid}$ and then explore whether any physically realistic solutions could be obtained.

### Alternate analysis approaches for determining slip lengths and/or apparent slip lengths

The results of the QCM data analyses were compared with two independent approaches for estimating slip lengths. The first relates the contact angle *θ* of a liquid atop a substrate to its predicted slip length^27^, and the second estimates the magnitude of the slip length that would be inferred from apparent slippage associated with reduced fluid density levels near the boundary^27,48–50^.

For the contact angle method, we utilized the parameters for water (*σ* = 0.276 nm, *r* = 0.385 nm) and also the relation reported by Ellis *et al*.:^48^

9 $$\frac{{\lambda }_{s}}{r}=\exp [\frac{\alpha A{\gamma }_{lv}(1-cos\theta )}{{k}_{B}T}]-1,$$

where the term *αA* is on the order of *σ*^2^, the molecular diameter squared, *r* is the center to center distance between molecules in the sheared liquid and *γ*_*lv*_ is the liquid vapor surface tension^27,48,49^. This approach, first suggested by Tolstoi and later extended by Blake^50^, is based on an observation that mobility of the liquid molecules adjacent to a solid surface should be linked to the equilibrium contact angle. In particular, the theory suggests that liquid molecules immediately adjacent to a surface will have the same mobility as the bulk for the case of complete wetting (*θ* = 0°) and mobility greater than the bulk for the case of partial wetting (*θ* > 0°). The enhanced mobility associated with non-zero contact angle in turn would give rise to a non-zero slip length. The method provides a means to independently and qualitatively rank the magnitude of the slip lengths in the systems studied, but has been found to consistently underestimate slip lengths^48–50^. Slip lengths obtained from analysis of the QCM data recorded here are therefore expected to have the same rank ordering, but different magnitudes from those obtained from Eq. (9).

The response of a QCM to the true slip is identical to that for the systems with density variations within the penetration depth. For a thin liquid film whose three-dimensional density is *ρ*_*film*_ and whose three-dimensional viscosity is *η*_*film*_, the slip length appears to be:^27^

10 $$\frac{{\lambda }_{s}}{\delta }=(\frac{\eta }{{\eta }_{film}}-\frac{{\rho }_{film}}{\rho }),$$

even in cases where there is no actual slippage. The QCM analyses were therefore also compared with Eq. (10) values under the assumption of a nanoparticle free region of pure water adjacent to the surface by setting *η*_*film*_ and *ρ*_*film*_ equal to the values for pure water, and *η* and *ρ* equal to the values of the bulk suspensions.

## Results and Discussion

### Contact angle and AFM surface roughness measurements

**Figure 3:**
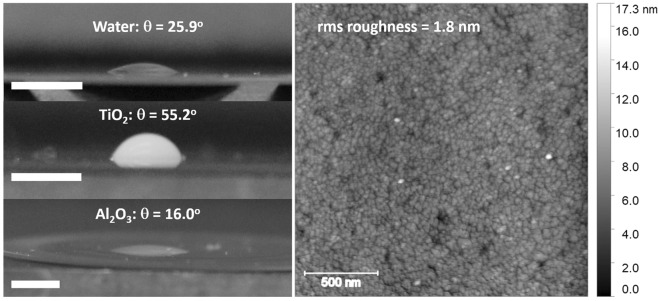
(Left) Photographic images of sessile droplets of (upper) pure water, and 0.67 wt% suspensions of (middle) TiO_2_ and (lower) Al_2_O_3_ atop the QCM platinum electrode, each with a scale bar of 0.3 cm. (Right) An AFM image of the surface topography of the electrode. For all Pt surface electrodes, both before and after an exposure to TiO_2_ and Al_2_O_3_, the rms roughness was 1.8 ± 0.2 nm, the fractal dimension was 2.54 ± 0.1, and the correlation length was 110 ± 10 nm.

Figure 3 (left) displays photographic images of the droplets and the contact angles measured for the deionized water and 0.67 wt% nanoparticle suspensions on Pt surfaces along with an AFM image (right) of the Pt QCM electrode. The TiO_2_ suspension exhibits a significantly higher contact angle, indicating that a considerably larger slip length is to be expected under the zero field conditions for this sample^48^. Using the experimental values for the contact angles the estimated slip lengths from Eq. (9) were found to be *λ*_s_ = 0.02 nm, 0.06 nm and 0.32 nm for Al_2_O_3_, water, and the TiO_2_ suspensions respectively.

The values for the saturated AFM roughness is *σ*_rms_ = 1.8 nm, with a lateral correlation length of 110 nm. This places the sample in the “slight” roughness regime categorized as σ_rms_/δ≪1. The response of the QCM is therefore theoretically predicted to be very close to that given by Eq. (4) when immersed in water^27^.

### QCM response in zero, static, and varying external electric fields

**Figure 4:**
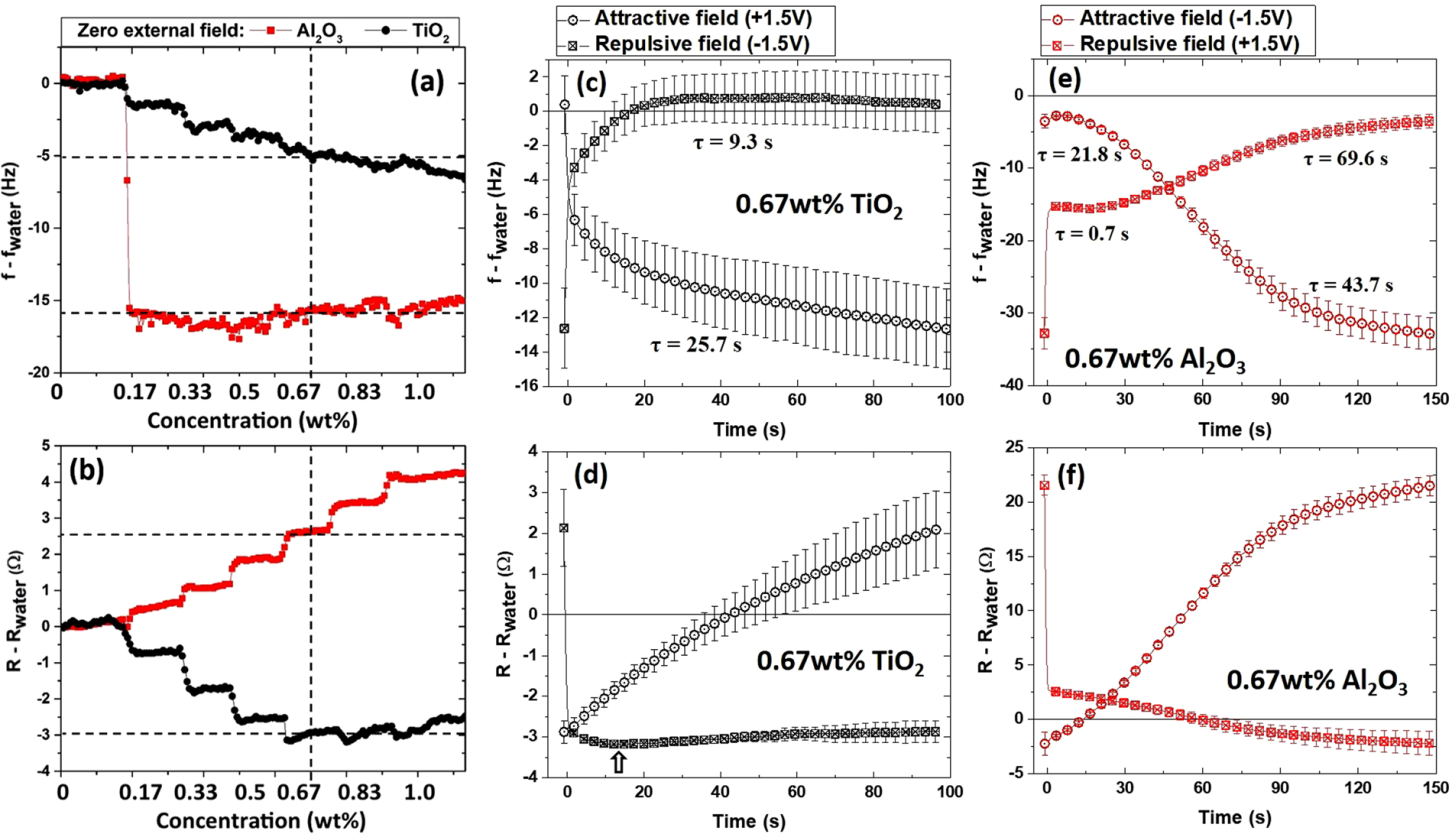
QCM frequency f (**a**) and resistance R (**b**) versus time as the concentration of positively (negatively) charged Al_2_O_3_ (TiO_2_) nanoparticles is increased from 0 to 1 wt% in six equal increments. TiO_2_ nanoparticles notably reduce frictional drag forces (R-R_water_) while Al_2_O_3_ nanoparticles increase them. (c-f) QCM frequency (**c**,**e**) and resistance (**d**,**f**) versus time for static electric field conditions for 0.67 wt% nanoparticle suspensions, where t = 0 is the time at which the applied squared wave bias voltage was reversed (once per 150s for Al_2_O_3_ suspensions and once per 100s for TiO_2_ suspensions). Error bars represent the standard deviation of five separate measurements.

**Figure 5:**
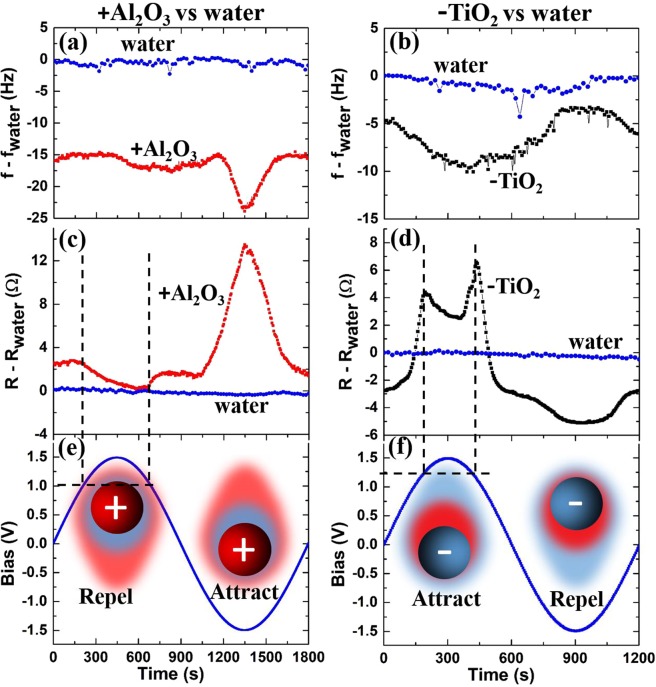
QCM frequency (**a**,**b**) and resistance (**c**,**d**) response for 0.67 wt% suspensions of Al_2_O_3_ and TiO_2_ nanoparticles and pure water (blue) for one cycle of applied alternating electric field (e,f). The features denoted by dashed lines (**c**–**f**) in between 1 and 1.25 V and may indicate TiO_2_ nanoparticles being pressed into interfacial water molecules, which also reorient at positive applied bias voltages.

Figures 4 and 5 display QCM frequency and resistance data for concentrations ranging from 0 to 1 wt% under zero external field conditions (Fig. 4a,b), for attractive and repulsive external fields applied to 0.67 wt% suspensions of TiO_2_ (Fig. 4c,d) and Al_2_O_3_ (Fig. 4e,f), and for slowly varying electric fields applied to the same suspensions (Fig. 5). The zero-field data were recorded upon immersing the electrode in water – the conditions under which platinum surfaces develop a negative charge^51^; therefore, the interfacial properties for the oppositely charged suspensions are expected to vary significantly from each other. The QCM data sets are indeed very different for the oppositely charged NP’s without an external electric field present, and are also quite responsive to the presence of the field. Overall, the nanoparticle systems behave as anticipated for attractive and repulsive fields: the QCM parameters trend in opposite directions under attractive and repulsive fields (Fig. 4c–f). Time constants displayed in Fig. 4(c–f) are obtained from fitting the data to an exponent approaching a limiting value. The motion of nanoparticles towards the surface is slower than the motion away from the surface. This is consistent with slowing of the Brownian motion of nanoparticles near surfaces^52,53^.

It is worthwhile to note here that the QCM data are not described by variations in concentration of the surrounding fluid under the no-slip conditions, Eq. (4). In this scenario the zero-field resistance (frequency) data (Fig. 4a,b) would monotonically increase (decrease) with the nanoparticle concentration. The resistance data for TiO_2_ actually go in the opposite direction when the particles are added, thus, clearly violating Eq. (4). Variations in concentration also fail to explain the alternating field data (Fig. 5), which displays distinct features associated with approach and retraction of the particles. The results as a whole are attributable to a complex interface, where changes in suspension density, particle uptake, interfacial slippage and friction levels, as well as changes in the slip plane location and the electric double layer configuration collectively contribute to the QCM response.

We begin the data analysis by comparing the zero field response to attractive and repulsive electric field conditions for 0.67 wt% suspensions, the nanoparticle concentration producing the greatest reduction in frictional drag forces for the TiO_2_ suspension. The dashed lines in Fig. 4(a,b) mark the frequency and the resistance response at 0.67 wt%, and fall close to (−15 Hz, +2.5 Ω) and (−5 Hz, −2.75 Ω) respectively for Al_2_O_3_ and TiO_2_. The average of multiple runs, along with the error bars, are depicted in Fig. 4(c–f), with the zero field average coinciding with the values marked in Fig. 4(a,b). The average values for an immersion in pure water were −727 Hz and +329 Ω. Frequency and resistance shifts for the suspensions relative to air under zero field conditions therefore respectively totaled (−742 Hz, 331.5 Ω) and (−732 Hz, 326.25 Ω) for Al_2_O_3_ and TiO_2_. Values for the suspensions exposed to constant attractive and repulsive fields after 100 s are also listed in Table 2. The corresponding real and imaginary components of the acoustic impedance, along with other relevant parameters are also listed in Table 2. Equations (1),(2) and (4) were used to obtain the proportionality constant between *R*_exp_(Ω) and ${\mathcal R} \,$_exp_(g cm^−2^s^−1^), employing the experimentally measured value of ${\mathscr{X}}$ for water obtained from the frequency shift data and Eq. (1). The measured frequency shift of −727 Hz for pure water was 6.3% higher than the theoretical value for a planar surface, −684 Hz (Eqs. 1 and 4, using the values in Table 1), an increase that is commonly reported in the literature and attributed to the surface roughness^29^. There is, however, no unified theory predicting the overall system response to roughness for both ${\mathscr{X}}$_fluid_ and ${\mathcal R}$_fluid_. We therefore assumed a universal 6.3% increase for the Table 2 values for both, i.e. ${\mathscr{X}}$_fluid_ = ${\mathscr{R}}$_fluid_  $\equiv 1.063\cdot \sqrt{\pi {\rho }_{s}{\eta }_{s}\,f}$, where *ρ*_s_ and *η*_s_ are respectively the density and viscosity of the fluid or suspension.

**Table 2:** Experimental QCM response to immersion into DI water and 0.67 wt% suspensions of TiO_2_ and Al_2_O_3_ in zero field and 100 s after switching between attractive or repulsive electric fields.

		DI water	Al_2_O_3_ suspension (positive charge)			TiO_2_ suspension (negative charge)		
		No field dependence	0 field	Attract: −1.5 V	Repel: + 1.5 V	0 field	Attract: + 1.5 V	Repel: −1.5 V
1	δ*f*_exp_ (Hz)	−727	−742	−756	−732	−732	−740	−727
2	δ*R*_exp_ (Ω)	329	331.5	348	326.5	326.25	331.2	326.1
3	δ*R*_exp_/δ*R*_water_	1	1.008	1.058	0.992	0.992	1.007	.991
4	${\mathscr{X}}\,$_fluid_ $= {\mathcal R} \,$_fluid_(g cm^−2^s^−1^)	405.3	407.2	407.2	407.2	407.5	407.5	407.5
5	${\mathscr{X}}\,$_exp_(g cm^−2^s^−1^)	405.3	413.5	421.2	407.9	407.9	412.4	405.1
6	${\mathcal R} \,$_exp_(g cm^−2^s^−1^)	405.3	408.4	428.7	402.2	401.9	408	401.7
7	δ${\mathcal R}$_exp_/δ${\mathcal R}$_fluid_	1	1.003	1.053	0.988	0.986	1.001	0.986

Acoustic impedance values have uncertainty ±1 g cm^−2^s^−1^

The QCM data presented in Table 2 reveal several interesting features. One notable observation is the similarity of the QCM response for TiO_2_ suspensions in an attractive field with Al_2_O_3_ data under zero field conditions (light grey columns). Data for the Al_2_O_3_ in repulsive fields are similarly comparable to TiO_2_ data recorded under zero field conditions (dark grey columns). Since the Pt surface at normal pH would have a negative charge without any applied external field^51^, the qualitative implication is that the repulsive field lifts Al_2_O_3_ particles from the electrode surface reaching a condition that is very similar to the TiO_2_ particles in the absence of such a field. Directing the TiO_2_ particles towards the surface by an electric field meanwhile results in a QCM response similar to that of Al_2_O_3_ in the absence of the field. Additional features in the Fig. 5 data might then be attributed to the physical properties of interstitial water layers present between the nanoparticles and the Pt substrate, or a lack thereof if the double layer does not remain intact.

The data reported in Table 2 were next compared to the limiting models of Fig. 2 by substituting the experimental parameters into Eqs. 4, 6, 7 or 8. Some of the models were found to be in clear contradiction with the experimental parameters while others allowed for the equations to be solved with reasonable physical parameters. Table 3 summarizes the results of substituting the Table 2 data in cases where the data did not contradict the models. All Table 2 data, with the exception of Al_2_O_3_ system in attractive field conditions, contradicted the Eq. 4 non-slipping bulk suspension model (Fig. 2b), which requires ${{\mathscr{X}}}_{{\rm{fluid}}}={ {\mathcal R} }_{{\rm{fluid}}}$. For the Al_2_O_3_ system in attractive fields the model predicts an unrealistic increase in the suspension concentration (~8 wt%). All of the Table 2 data contradicted the slipping bulk suspension model (Fig. 2c). Two systems, Al_2_O_3_ in the zero field and TiO_2_ in an attractive field, yielded realistic solutions to the no-slip film and no-slip bulk suspension model (Fig. 2d). All other Table 2 data contradicted the model. Three systems, TiO_2_ in the zero field and both Al_2_O_3_ and TiO_2_ in attractive fields, yielded realistic solutions to Eq. 7 with a >0, the no-slip film with slipping bulk suspension model (Fig. 2e). Equation 8 corresponding to the slipping adsorbed film model could also be solved for the three systems (Fig. 2f) but only one of these, the Al_2_O_3_ suspension under attractive field conditions, yielded physically realistic parameters. Two others systems, Al_2_O_3_ in the zero field and TiO_2_ in an attractive field, yielded solutions corresponding to only trace levels of the film uptake. These were ruled out as physically unrealistic since the model assumes a dense film is present.

**Table 3:** Solutions to the various models’ equations employing the data reported in Table 2.

Non-slipping bulk (Eq. 4): The model predicts an unrealistic increase in Al_2_O_3_ suspension concentration (~8 wt%) for −1.5 V attractive fields. All other Table 2 data contradict this model.							
Slipping bulk suspension (Eq. 6): All Table 2 data contradict this model.							
Non-slipping bulk/non-slipping film model (Eq. 7 with a = 0): The model predicts realistic solutions for the 2 systems listed below. All other Table 2 data contradict the model.							
**Sample**	**Bias** **V**	***a***	***λ***_***s***_ **nm**	***η***_**2**_ **g cm**^**−2**^**s**^**−1**^	***ρ***_**2**_ **μg cm**^**−2**^	***ρ***_***2***_ **NP monolayers**	**τ** **ns**
Al_2_O_3_	0	0	0	-----	0.16	0.02	0
TiO_2_	+1.5	0	0	-----	0.14	0.01	0
Electrolyte model (Eq. 7 with a > 0): The model predicts realistic solutions for the 3 systems listed below. All other Table 2 data contradict the model.							
Al_2_O_3_	+1.5	0.085	20.7	4500	2.0	0.285	0.45
TiO_2_	0	0.090	21.9	4300	2.2	0.210	0.50
TiO_2_	−1.5	0.092	22.3	4200	2.2	0.205	0.50
Adsorbed film model (Eq. 8): The model predicts one realistic solution for Al_2_O_3_ under attractive field conditions. All other Table 2 data contradict the model.							
Al_2_O_3_	−1.5		30	3100	4.96	0.7	1.6
**Sample**		**Contact angle method** ***λ***_***s***_***(nm)***	**Variable density model** ***λ***_***s***_***(nm)*** Eq. (10)				
+Al_2_O_3_		0.02	2.08				
−TiO_2_		0.32	2.40				

Slip lengths for the three systems that matched the slipping film-fluid boundary model are in the range of 20–22 nm. This is greater than the slip length that would arise from a density variation artifact associated with the pure water near the surface (Table 3, variable density model), and is unlikely to be an artifact. The values are meanwhile close to the slip lengths reported in literature^48,54,55^. The slip times obtained for these systems are of the order 10^−10^ s, which is also physically realistic as the typical values range from 10^−12^ to 10^−8^ s^46^. A slip length of 30 nm was obtained for the Al_2_O_3_ under the attractive field conditions employing the “adsorbed film” model that attributes all increases in resistance to a slippage at the film-substrate boundary.

One possible interpretation of the results obtained with these models is that an interstitial layer of water is present between the TiO_2_ particles and the substrate but not the Al_2_O_3_ layers under zero field conditions because the negative charge on both the NP and the substrate would act to stabilize the double layer. As the TiO_2_ (Al_2_O_3_) particles are directed into (or retracted from) the surface, water might be squeezed out (or reformed), resulting in additional non-monotonic features in the data associated with the physical properties of the confined molecules^54,56–59^. No-slip boundary conditions and/or high interfacial friction levels would then be linked to the absence of the interstitial water layer(s). This interpretation is consistent with fact that some fine features is observed in the data for the TiO_2_ suspensions for both retraction and approach, but only for the Al_2_O_3_ suspensions in retraction. The features in the data are not readily attributable to electrolysis, as no bubbles were observed either visually or in a form of the characteristic QCM response in water or the nanoparticle suspensions themselves. It is known, however, that the voltage levels corresponding to the electrolysis are closely linked to the thickness of the oxide layers on the platinum surface and significantly increase the voltage threshold at which the steady-state electrolysis first occurs^60,61^. It is also well known that water molecules can reorient and also solidify under the influence of external fields. The latter phenomenon is expected to have a great impact on the interfacial friction^58^.

In summary, although the origins of the physical phenomena reported here have yet to be fully illuminated, the results are reminiscent of the variations in friction observed in SFA as molecular layers are squeezed out from an interface^54^. Numerical simulations of nanodiamond systems with positive and negative charge have revealed the presence of the interstitial water layers^56^, but simulations of the present systems under tunable external electric fields remain to be reported. Such computational studies would be also highly valuable for further development of this method. The present results nonetheless demonstrate the sensitivity of the QCM technique to identify molecular repositioning near interfaces and suggest that the charged nanoparticles can be actively repositioned to explore interfacial properties and nanoscale interactions in geometries inaccessible to optical and micromechanical probes.

## Data Availability

The datasets generated during and/or analyzed during the current study are available at 10.7910/DVN/YRTIM1.
